# Brain Responses to Peer Feedback in Social Media Are Modulated by Valence in Late Adolescence

**DOI:** 10.3389/fnbeh.2022.790478

**Published:** 2022-05-30

**Authors:** Patrik Wikman, Mona Moisala, Artturi Ylinen, Jallu Lindblom, Sointu Leikas, Katariina Salmela-Aro, Kirsti Lonka, Berna Güroğlu, Kimmo Alho

**Affiliations:** ^1^Department of Psychology and Logopedics, Faculty of Medicine, University of Helsinki, Helsinki, Finland; ^2^Advanced Magnetic Imaging Centre, Aalto NeuroImaging, Aalto University, Espoo, Finland; ^3^Faculty of Social Sciences, University of Tampere, Tampere, Finland; ^4^Department of Clinical Medicine, University of Turku, Turku, Finland; ^5^Swedish School of Social Science, University of Helsinki, Helsinki, Finland; ^6^Faculty of Educational Sciences, University of Helsinki, Helsinki, Finland; ^7^School of Education, Michigan State University, East Lansing, MI, United States; ^8^Optentia Research Focus Area, North-West University, Vanderbijlpark, South Africa; ^9^Institute of Psychology, Developmental and Educational Psychology Unit, Leiden University, Leiden, Netherlands

**Keywords:** brain, social media, feedback, peers, late adolescence, fMRI

## Abstract

Previous studies have examined the neural correlates of receiving negative feedback from peers during virtual social interaction in young people. However, there is a lack of studies applying platforms adolescents use in daily life. In the present study, 92 late-adolescent participants performed a task that involved receiving positive and negative feedback to their opinions from peers in a Facebook-like platform, while brain activity was measured using functional magnetic resonance imaging (fMRI). Peer feedback was shown to activate clusters in the ventrolateral prefrontal cortex (VLPFC), medial prefrontal cortex (MPFC), superior temporal gyrus and sulcus (STG/STS), and occipital cortex (OC). Negative feedback was related to greater activity in the VLPFC, MPFC, and anterior insula than positive feedback, replicating previous findings on peer feedback and social rejection. Real-life habits of social media use did not correlate with brain responses to negative feedback.

## Introduction

A great deal of our waking hours is spent in front of a screen, most often on various social media platforms. A national American survey revealed that adolescents ages 13 to 18 spend around 6–7 h in front of a screen during their free time (Rideout, 2015), and according to a 2018 report, 45% of American teens say they use the internet “almost constantly” (Anderson and Jingjing, 2018). This trend continues past the teenage years, as according to a report from the United Kingdom, young adults ages 18 to 24 spend an estimated 4 h per day on their smart devices (Ofcom, 2018). The most popular online platforms among teens and young adults are YouTube, Instagram, Snapchat, and Facebook (Anderson and Jingjing, 2018; Smith et al., 2018), which are all social in nature. Further, some recent research has shown that excessive social media use is associated with increase in mental health problems, and cybervictimization in adolescents. Given how much time especially young people spend on social media, it is crucial to understand the social, emotional and neurocognitive underpinnings of this highly prevalent daily activity. One way to approach this topic is to examine how the emotion-evoking aspects of social media are associated with brain activity, and whether individual differences play a role in neural responses to the affective content of virtual social interaction.

Virtual social interaction has often been studied in adolescents and young adults, because the vast majority of young people’s daily smartphone use is related in some way to social interaction and connection (Anderson and Jingjing, 2018; Smith et al., 2018), and the socio-cognitive functions undergo substantial neural development during the adolescent years. These two phenomena may be linked, so that the fact that young people’s online activity is predominantly social may, in part, be explained by maturation that occurs specifically in adolescence and young adulthood in brain networks involved in social behavior. For example, during adolescence the brain shows heightened sensitivity and responsivity to social feedback from peers (Burnett et al., 2011). Regions related to mentalizing and perspective taking in the medial prefrontal cortex (MPFC) also mature during this time (Blakemore, 2008). Given these age-related changes in the “social brain,” young people may be especially sensitive to the emotional content of social media (Crone and Konijn, 2018). Social media platforms offer an opportunity to receive peer feedback at a high frequency, and this feedback strongly activates brain areas involved in socio-emotional processing (Sherman et al., 2018).

Two of the most studied valenced aspects of virtual social interaction are peer feedback and social rejection. Although these phenomena are usually studied separately, they are partly overlapping. That is, receiving negative feedback from a group of peers is likely to elicit feelings of being ostracized or rejected. Multiple experimental paradigms have been designed to study how the brain reacts to receiving positive or negative feedback from peers in a virtual environment. These paradigms have utilized tasks that aim to imitate aspects of real-world social media platforms. For example, in the Ostracism Online task, participants join an online group, introduce themselves, and get a variable amount of likes from other participants (Wolf et al., 2015). Receiving few likes in this task has been shown to lead to a negative emotional state, decreasing feelings of self-esteem and belongingness (Schneider et al., 2017). In a similar vein, Somerville et al. (2006) devised a task where participants were shown facial images of strangers and were asked to rate their likeability. Participants then received either positive or negative feedback to their own picture from these individuals. Brain activity recordings during this task showed that the ventral portion of the anterior cingulate cortex (ACC) was differentially responsive based on the valence of the feedback (i.e., greater activity for positive feedback), supporting previous findings linking the ventral ACC to social and emotional evaluation. A modified version of this task was used to study age-related changes in neural reactions to peer feedback in a sample of 8–25-year-olds (Gunther Moor et al., 2010). The study revealed an age-related linear increase for activation in the striatum, MPFC, orbitofrontal cortex (OFC), and lateral prefrontal cortex (LPFC) for rejection feedback, with no differences between the age groups noted for positive feedback. The authors suggested that heightened activity in these regions in older participants reflected more mature affect regulation and self-control. Achterberg et al. (2016), in turn, used a virtual social judgment paradigm where participants received positive or negative peer feedback based on their profile picture. They noted increased activity in the insula and MPFC during both positive and negative peer feedback. This was interpreted to reflect the engagement of the brain’s saliency network due to detecting socially relevant events.

When studying virtual ostracism, a widely used task has been the Cyberball paradigm, where participants play a virtual ball-tossing game with two other players and are either included or ignored (Williams et al., 2000). This paradigm has revealed that adolescents react more strongly to peer rejection than adults, as indicated by a lower mood and higher anxiety after being ostracized in the task (Sebastian et al., 2010). Not all studies have replicated this finding, however, (e.g., Gunther Moor et al., 2012). Combining the Cyberball paradigm with neuroimaging, Masten et al. (2009) found that during ostracism, adolescents display activity especially in the anterior insula and right ventrolateral prefrontal cortex, likely reflecting emotional distress and the need to regulate negative affect. In a similar vein, in their meta-analysis of studies in adults, Cacioppo et al. (2013) found that the feeling of being ostracized in the Cyberball game increases activity in the OFC, anterior cingulate, and anterior insula, possibly reflecting social rumination and distress. The study of Gunther Moor et al. (2012), in turn, revealed a network of brain regions in the MPFC, LPFC, ACC, and anterior insula that was more responsive to exclusion than inclusion in the Cyberball task in a sample of 10–21-year-olds. The only result concerning age-related differences in the study was that 10–12-year-olds, but not the older participants, showed increased activity in the ACC during social exclusion.

As described above, virtual social interactions have been studied using a variety of different paradigms. However, these paradigms do not necessarily correspond well with adolescents’ experiences in their real-life social media environments. Such correspondence would be critically important when analyzing the effects of social media use habits on brain activity during virtual interactions. Thus, we propose that utilizing familiar social media platforms and emulating naturalistic peer responses will help to determine how the findings from previous brain studies using less-naturalistic tasks generalize to real-life social media behavior.

Interestingly, systematic individual differences in neural responses to social feedback and rejection have also been reported. For example, in the study of Gunther Moor et al. (2010), higher levels of social anxiety and self-perceived self-worth were linked to larger activation in the subcallosal cortex, paracingulate cortex, OFC, LPFC, and putamen during social rejection when having a negative expectation of the social evaluation. This was thought to reflect a stronger impact of social rejection feedback in these individuals. In a similar vein, it has been found that individuals with lower levels of social support display higher levels of activity in brain regions related to emotional processing during virtual social exclusion (Eisenberger et al., 2007; Masten et al., 2012).

The current study aimed to test whether the same neural networks previously associated with virtual social interaction are activated using a new naturalistic paradigm that incorporates a Facebook-like platform familiar to adolescents and young adults. We expected to replicate the previous findings showing enhanced activity in the insula and MPFC in response to emotion-evoking peer feedback, as well as activity in the ACC, LPFC, and anterior insula specifically in response to negative peer feedback and social rejection. Moreover, we explored whether there is a relationship between individual brain reactivity to peer feedback and habits of social media use. For example, a tendency to react more strongly on a neural level to social rejection might be a reason to avoid social media (i.e., spend less time on social media and have fewer social media contacts). We assessed brain activity with functional magnetic resonance imaging (fMRI) while the participants were engaged in a task on a Facebook-like platform requiring them to give their opinions on controversial statements and received mock positive and negative feedback on their opinions from their peers. The brain responses to peer feedback were examined both independently and in relation to background variables of participants’ social media use.

## Materials and Methods

### Participants and Procedure

In total, data from 95 voluntary participants were analyzed. The participants were obtained from two different research projects (Sample 1: 26 participants from the project *Bridging the Gaps – Affective, cognitive, and social consequences of digital revolution for youth development and education*, University of Helsinki; Sample 2: 69 participants from the project *Miracles of Development*, Tampere University). Both samples are prospective community samples. We collected the demographic variables of age and gender and level of education. The mean age in Sample 1 was 18.1 years, and the mean age in Sample 2 was 19.0 years. That is, the participants in the two samples differed in age [*t*(90) = −5.59, *p* = 2.51e^–7^]. The gender distribution in the first sample (Gaps) was 39% female and 61% male, and in the other (Miracles) 64% female and 36% male. The gender distribution significantly differed in the two samples (χ^2^ = 4.8, *p* = 0.028). All participants (except two participants whose background information was missing; Sample 1) from both samples were completing or had just completed secondary school at the time of data collection.

To justify our sample size, we performed power analyses based on prior literature. The present sample size was deemed sufficient for the planned analyses. Regarding the within-participant analysis, previous fMRI studies on neural correlates of virtual social feedback and exclusion have used sample sizes of ca. 15–60 participants (e.g., Somerville et al., 2006; Gunther Moor et al., 2010; Cacioppo et al., 2013; Achterberg et al., 2016). For example, Gunther Moor et al. (2010) reported significant clusters for a contrast between like vs. dislike judgments on profile picture, for a group of 16 adolescents (uncorrected *p* < 0.001). Given this p-threshold and the degrees of freedom in their study, the smallest effect size to survive this threshold was calculated to be Cohen’s *d* = 0.925. In the current study, we chose to be conservative so we to used family-wise correction on the voxel level for our within-participant analysis, thus with 46,082 voxels, our family-wise corrected p threshold would be 0.05/46082 = 1.08 × e^–6^. Given this alpha level, the expected effect size of 0.92, 48 participants would be necessary to yield an 80% power, and 70 participants would be needed to yield a 99% power (calculated using G-power; Faul et al., 2007) for a similar contrast. Between-participant analyses of brain imaging data often entail smaller effect sizes. In our previous studies (Moisala et al., 2016, 2017), we observed effect sizes in the order of η2 = 0.03–0.04 for associations between individual background variables and brain activity in region-of-interest (ROI) analyses. Effect sizes of this magnitude require an estimated sample size of ca. 100 participants to achieve a statistical power level of 0.8 at a alpha level of *p* < 0.05 (calculated using G-power). Previous studies examining individual differences in neural sensitivity to peer rejection have used sample sizes of ca. 20–50 participants (Masten et al., 2012; Will et al., 2016).

Data from three participants had to be discarded due to excessive head movements during fMRI scanning. These participants, all from Sample 2, were identified using a cut-off value of >0.19 mm for mean framewise displacement (Power et al., 2014), corresponding to two standard deviations from the mean of all participants. The remaining 92 participants (52 females) were 17 to 20 years old (*M* = 18.70, SD = 0.72). They were all native Finnish speakers with normal hearing, normal or corrected-to-normal vision, and no self-reported history of psychiatric or neurological illnesses. Informed written consent was obtained from each participant before the experiment. The experimental protocol was approved by the Ethics Committee of The Hospital District of Helsinki and Uusimaa.

The participants were introduced to the experimental task by telling them that they would be posting opinions in a Facebook “group” created by the experimenters, and receiving peer feedback to those posts from peers. Although our previous results indicated that neural activation patterns would be stronger if the feedback was coming from personally familiar peers as opposed to the unfamiliar peers (Güroğlu et al., 2008), we chose to use unknown peers, as the participants were then less likely to doubt the authenticity of the feedback. Facebook as a social media platform was familiar to all participants, and 90.2% of the participants reported currently having a Facebook account. The participants were told that the peer feedback to their posts had been written by peers who had been recruited for the study and asked to write their honest opinion on the statements. In an initial pilot testing phase, the participants were told that the peer feedback was written in real-time from remote stations, but this did not seem credible due to the relatively fast rate at which the comments appeared. Since the latency of the peer feedback could not be delayed due to restrictions on measurement time, the task instructions were modified in the final study. The participants were now told that the peer feedback consisted of genuine reactions from peers to the presented opinions, but that these reactions had been prerecorded (for each possible opinion), and were presented to the participants at a faster rate than usual due to time restrictions. After completing the task with these modified instructions, only 16% of the participants included in the current study’s analyses expressed doubts about the credibility of the task in a post-task interview.

The participants rehearsed the task on a computer in a separate room before entering the scanner to complete the actual experiments. During their time in the MRI scanner, they performed also other experimental tasks, to be reported elsewhere, and in total, they spent approximately 1 h in the scanner and 1 h preparing for the study. In addition to the task of the present study, the participants belonging to Sample 1 performed a speech-listening and reading task requiring selective and divided attention to written and spoken sentences (described in detail in Moisala et al., 2016). This task was practiced before the fMRI scans, but within the fMRI session, the sentence task was performed after the social media task of the present study. The participants belonging to Sample 2, in turn, performed two additional tasks, both of which were performed in the scanner before the social media task of the present study. With these participants, an anatomical scan (MPRAGE) was also obtained between the two additional tasks and the present task. The first of the additional tasks (adapted from Hare et al., 2008) was a go/no-go task requiring the participants to monitor a stream of photographs of faces and respond to them based on their facial expressions (happy, angry, or neutral). The other additional task (adapted from Gunther Moor et al., 2012) involved photographs of faces cropped to show only the eyes. This task included two sub-tasks, in which the cropped face was first shown and the participants then had to pick a word to either describe (1) what the person in the photograph was thinking about or feeling, or (2) the gender and age of the person in the photograph. The participants were reimbursed 15 €/hour for their time (2–3 h). Due to the emotional nature of the present task, a trained staff member debriefed all participants after the study.

### Task and Stimuli

**FIGURE 1:**
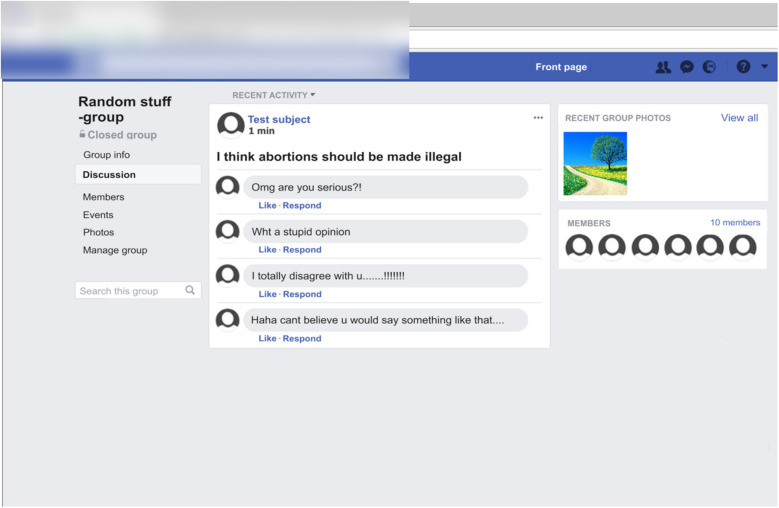
An example screenshot (translated from Finnish to English for descriptive purposes) of the mock Facebook environment used in the study. The response of the participants to a controversial statement is presented as a “post” in a generic Facebook group. The post is followed by four comments from perceived peers. In this example, the participant has responded with “agree” to a controversial statement and has received negative peer feedback to their “post” from peers.

The main task of the participants was to respond with a button press to short statements presented on a Facebook-like computer scene and then view peer feedback to a post generated based on their responses to the statements from peers in a simulated social media environment. The statements (written in Finnish) were controversial (e.g., “*Abortions should be illegal,” “Fur-farming is unethical,” “Men are smarter than women,”* etc.), and the participants were asked to respond by pressing either a “agree” button or a “disagree” button to each statement (see Supplementary Table 2 for the translated statements). The statements were purposefully controversial to make the task engaging and to evoke interest and emotional involvement in the peer feedback following the statements. However, they included a range of different topics. The participants’ response was presented in a generic-looking Facebook “group” as a “post” presenting their opinion on first-person form (e.g., *“I think abortions should be legal”*). The mock Facebook environment was created by taking a screenshot of the main page of a real Facebook group, and by editing it to look like a generic Facebook group (Figure 1). Each post was followed by four comments (in Finnish) from peers. The peer feedback to each post were either positive (e.g., “*I totally agree with you.*”), or negative (e.g., “*What a stupid opinion!”*) in nature. The peer feedback was pre-generated by the experimenters but were designed to seem as if they were authentic comments from peers (i.e., they included spelling mistakes and colloquialisms).

As a control task, participants were presented with neutral, factually correct statements (e.g., “*Helsinki is the capital of Finland*”; for all statements, see Supplementary Table 2) and asked to respond to each statement by pressing either a “true” or “false” button. Similar to the controversial statements, the neutral statements were also then presented in the mock Facebook “group” as a post, but without modifying them into the first-person form. Neutral statements were not followed by peer feedback, but by four neutral statements concerning the topic of the post (e.g., “*The city of Helsinki was founded in 1550*”) that the participants were told had been written by the experimenters. Thus, the control task was visually, linguistically and motorically similar to the main task, but it lacked the components of emotional valence and peer feedback. In the control task, we chose to use factual statements that were obviously true (e.g., “The sun sets in the night,” see Supplementary Table 2), as we did not want participants to engage in more complex decision making between true and false statements.

The mean length between neutral statements and valenced statements did not differ significantly from each other [respective mean lengths ± SD: 32.98 ± 6.42 and 31.29 ± 10.62; *t*(52) = 0.62, *p* = 0.54].

**FIGURE 2:**
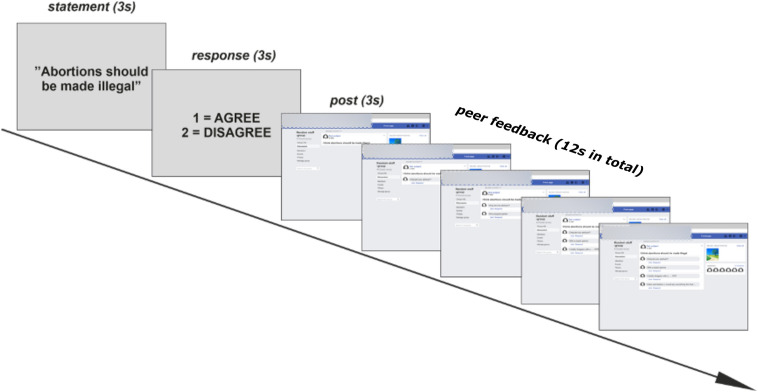
A schematic illustration (translated from Finnish to English) of the procedure of the study. In the sequence of an example trial, where the participants are presented with a controversial statement, they respond with “agree,” and then receive negative peer feedback from peers.

The structure of a task trial is demonstrated in Figure 2. Each trial began with the presentation of the written statement for 3 s, followed by a 3-s response window. For controversial statements, the response options were: “agree” (index finger) and “disagree” (middle finger). For neutral statements, the response options were: “true” (index finger) and “false” (middle finger). The response window was then followed by a pre-recorded video presenting the statement as a “post” in a mock Facebook group. Each post was presented for 3 s. The post then remained on the screen while below the post appeared four “comments” from peers who were members of the mock Facebook group. The comments were posted successively, and they appeared at intervals of 2, 2.7, 3.3, or 4 s, in random order. Thus, the comments, appeared within a time window of 12 s after which the next trial began. The inter-trial interval was jittered between 3 and 7 s. During this interval, there was a fixation cross in the middle of the screen. At the end of each block, there was a 40-s block of rest with the fixation cross.

Each block consisted of 18 trials, six for each of the three conditions: (1) a neutral statement with neutral peer feedback, (2) a controversial statement with positive peer feedback, and (3) a controversial statement with negative peer feedback. To increase the credibility of the task, some neutral peer feedback was included among the positive/negative peer feedback to controversial posts. More specifically, in 50% of trials with controversial statements, one neutral comment was included, and in 33% of the trials, two neutral comments were included. Given that the comments were mostly in agreement with each other within a trial (i.e., there was never a mix of negative and positive comments to a “post”), it could be argued that the trials with (mostly) negative feedback task included aspects of both peer feedback and peer rejection.

The order of the trials in each block was randomized. Twenty-six of the participants (Sample 1) completed three functional blocks of the task, totaling 18 trials per each task condition and 225 volumes measured per block, resulting in 32 min of task in total. The remaining 66 participants (Sample 2) completed 2 blocks of the task, totaling 12 trials per condition with 215 volumes per block, resulting in 18 min of task in total. The differences in volumes per block were due to unequal amounts of dummy volumes (Sample 1 had 10 additional dummy volumes). Less data were collected from the latter group due to restrictions on total measurement time, as the group performed other tasks during the same measurement.

### Questionnaire Concerning Social Media Use

In addition to completing the neuroimaging task, during their visit to the laboratory the participants filled out a questionnaire concerning their habits of social media use. Three open-ended questions concerning social media use were used in the analyses of the current study: (1) “How many friends do you have on your favorite social media app that you are in active contact with on a weekly level?” (i.e., *Number of active social media contacts*), (2) “How many minutes do you spend on an average day on your favorite social media site?,” with values averaged across typical weekdays and typical weekends (i.e., *Time spent on social media*), (3) “When you add a photo of yourself on social media, how many “likes” do you typically get to that photo?” (i.e., *Popularity on social media*). When answering the questions related to social media use, participants were asked to estimate the answer based on their recollection.

Pearson correlation coefficients were calculated to check whether answers to the questionnaire variables varied significantly with age. Differences in the questionnaire variables between genders were studied by using independent-samples *t*-tests.

### Functional Magnetic Resonance Imaging Acquisition

Functional brain imaging was carried out with a 3 T MAGNETOM Skyra whole-body scanner (Siemens Healthcare, Erlangen, Germany) at the AMI Centre, Aalto NeuroImaging, Aalto University School of Science, Espoo, Finland. A 20-channel head coil was used. The functional echo-planar (EPI) images were acquired with an imaging area consisting of 43 contiguous oblique axial slices (TR 2,500 ms, TE 32 ms, flip angle 75°, voxel matrix 64 × 64, field of view 20 cm, slice thickness 3.0 mm, in-plane resolution 3.1 mm × 3.1 mm × 3.0 mm). Image acquisition was performed at a constant rate (i.e., image acquisition was not jittered), but was asynchronized with stimulus onsets. High-resolution 3D T1 anatomical images (voxel matrix 256 × 256, in-plane resolution 1 mm × 1 mm × 1 mm) were acquired from each participant in the task.

### Functional Magnetic Resonance Imaging Preprocessing and Analyses

Image preprocessing and statistical analysis were performed using Statistical Parametric Mapping (SPM12) analysis package (Wellcome Department of Cognitive Neurology, London, United Kingdom; Friston et al., 1994) as implemented in Matlab. In pre-processing, the slice timing was corrected, data were motion-corrected, high-pass filtered (cut-off at 1/128 Hz), and spatially smoothed with a 6-mm Gaussian kernel. The EPI images were intra-individually realigned to the middle image in each time series and un-warping was performed to correct for susceptibility artifacts and head movements.

For the first-level statistical analysis, the general linear model (GLM) was set up with separate regressors for each event type within a trial. These events were (1) The presentation of the statement (neutral or valenced), (2) the participant’s response (“true”/“false” or “agree”/“disagree”), and (3) the emotional tone of the peer feedback (positive/negative/neutral). We only defined contrasts for the statement and the feedback as this was the focus of the current paper and there were differences in behavioral responses between the conditions (see Table 1). Trials where no response was given by the participant were modeled with separate regressors covering the entire duration of the event and excluded from the contrasts of interest. All data and regressors were estimated within a single GLM. Separate regressors for the responses of the participants and instructions, as well as 6 movement parameters were added as nuisance regressors. The task-related regressors were convolved with the canonical hemodynamic response function.

**TABLE 1:** Percentages of different response types to neutral and controversial statements.

Statement type	Participant’s response	MEAN (%)	SD (%)
	*“true”*	91.89	10.42
Neutral	*“false”*	5.44	8.29
	no response	2.28	6.05
	*“agree”*	48.05	9.34
Valenced	*“disagree”*	47.73	11.02
	no response	4.01	6.85

In the second-level analysis, anatomical images were normalized to a canonical T1 template (MNI standard space) provided by SPM12 and then used as a template to normalize the functional images of each participant (tri-linear interpolation, 3 mm × 3 mm × 3 mm, using 16 non-linear iterations).

### Whole-Brain Analysis of Functional Magnetic Resonance Imaging Data

The first aim of the fMRI analyses was to determine whether our social media task elicited similar neural activity patterns as previous studies using more traditional experimental paradigms probing socio-emotional brain processes. To this end, whole-brain statistical maps were calculated for the contrast *valenced feedback* > *neutral feedback*. For comparison, whole-brain statistical maps were also calculated for the contrast *valenced statement* > *neutral statement*, as also this contrast was expected to reveal socio-emotional brain activity associated with decision making in a social context rather than social feedback. Further, we wanted to determine whether negative and positive peer feedback elicited significantly differing brain activations. Therefore the contrasts between *negative feedback > positive feedback* and *positive feedback > negative feedback* were calculated and overlaid on top of each other for visualization purposes.

The resulting statistical maps were voxel-level family-wise error corrected (*p* < 0.05), with a cluster size minimum of 100 voxels (see Griffis et al., 2016; Leminen et al., 2020). We chose a cluster size minimum of 100 voxels to limit false positives related to small clusters. If no clusters survived this more stringent correction, a cluster-level family-wise error correction (*p* < 0.05) was used, while the cluster size minimum was maintained at 100 voxels. The *t*-value height thresholds and cluster size thresholds are presented under each contrast image in the see section ‘‘Results.’’ Anatomical regions corresponding to the activity foci were identified using the xjView toolbox for SPM^1^.

### Region-of-Interest Analysis of Functional Magnetic Resonance Imaging Data

When studying how neural sensitivity to negative peer feedback was associated with social media use, analysis of mean signal changes was restricted to brain regions involved in the processing of peer feedback, as determined by the whole-brain analyses described previously. This method is considerably more powerful than doing whole brain regression analyses that require correcting for searching the whole volume, thus inflating the probability of Type II errors. More specifically, regions-of-interest (ROIs) were chosen based on the whole-brain analyses using the contrast *peer feedback > neutral feedback.* Since we aimed to specifically study reactivity to negative feedback, the mean % signal change for events with positive feedback was subtracted from the mean % signal change for events with negative feedback in these ROIs. This measure was then subjected to a mixed-measures analyses of variance (mixed ANOVAs). Three within-participant variables related to social media use were included in the mixed ANOVA: (1) *Number of active social media contacts*, (2) *Time spent on social media*, and (3) *Popularity on social media.* ROI was included as a within-participant variable to determine whether the background variables of interest were associated with activity levels in all ROIs (thus producing a main effect) or only in selected ROIs (thus producing an interaction effect with ROI). Age and gender were also included as covariates as they may affect fMRI results because neural maturation is thought to be ongoing within the age range of our sample (Tamnes et al., 2017) at a rate that varies between genders (Lenroot et al., 2007). We chose to do these analyses utilizing an ROI approach, since studying the effects related to social media use throughout the entire brain volume was deemed to be too insensitive, given that the effects of social media use on brain activity are likely to be moderate at best (see Moisala et al., 2016).

The ROI analysis of fMRI data was conducted using the MarsBaR toolbox^2^. Partial eta-squared (η_*p*_^2^) was calculated for each mixed ANOVA as a measure of effect size. For all mixed ANOVAs, the Greenhouse-Geisser *p*-value was used (as indicated in Results by an accompanying correction value ε) if Mauchly’s test of sphericity showed a significant result for a variable with more than two levels. However, even in these cases, original degrees of freedom will be reported with the *F*-value. A 95% confidence interval was used in all mixed ANOVAs. When a mixed ANOVA yielded a significant result, a multiple regression analysis was conducted to examine simple main effects. IBM SPSS Statistics 21 for Windows (IBM SPSS, Armonk, NY, United States) was used for statistical analyses.

## Results

### Social Media Use

The means and SDs of social media use variables are listed in Table 2. Included are also their associations with age as well as differences between genders.

**TABLE 2:** Means and standard deviations (SDs) of the social media use (separately for males, M; and females, F) variables, their associations with age, and their differences between genders.

Questionnaire variable	Mean (M, F)	SD (M, F)	Association with age	Difference between genders
*Number of contacts*	126.4, 212.3	117.1, 202.3	n.s.	n.s.
*Time spent (in minutes)*	140.0, 190.6	110.1, 106.4	n.s.	*p* < 0.05*
*Popularity (number of likes)*	112.1, 186.9	120.2, 107.4	n.s.	*p* < 0.005**

*Significant associations are bolded.*

No significant associations between age and the social media use variables were observed (Pearson correlation coefficients, *p* > 0.05). *Time spend on social media* (reported as minutes per day) was higher for female participants (mean ± SD: 190.59 ± 110.08) than for male participants (139.97 ± 106.36) with a statistically significant difference of 50.61 min/day (95% confidence interval, CI: 4.75–96.48), *t*(88) = 2.19, *p* = 0.03. *Popularity on social media* (reported as a typical number of likes per one’s own photo) was also higher for the female participants (186.92 ± 120.21) than for the male participants (112.14 ± 107.42) with a statistically significant difference of 74.78 likes (95% CI: 25.14–124.43), *t*(86) = 3.00, *p* = 0.004.

Correlations between the social media use variables are presented in Table 3. The number of social media contacts correlated significantly with popularity on social media, whereas time spent on social media correlated significantly positively with popularity on social media and negatively with emotional stability and openness.

**TABLE 3:** Correlations between the social media use variables.

	1	2	3
1. Number of contacts			
2. Time spent	0.22		
3. Popularity	0.37**	0.35**	

**p < 0.005. Significant associations are bolded.

The 16% of participants who expressed doubts about the credibility of the task did not differ statistically significantly in age, gender or social media use variables from those not expressing doubts. The participants belonging to the two samples included in the study did not differ significantly from each other on any of the social media use variables.

### Behavioral Results

The percentages of different response types given by the participants to neutral and controversial statements are presented in Table 1. We did not calculate mean reaction times per task as it would not adequately reflect task performance. This is because (1) participants had already made their decision when the prompt to answer was given; and (2) participants were allowed to answer with a long response window (3 s), i.e., very little time pressure after the prompt appeared.

### Functional Magnetic Resonance Imaging Results

**FIGURE 3:**
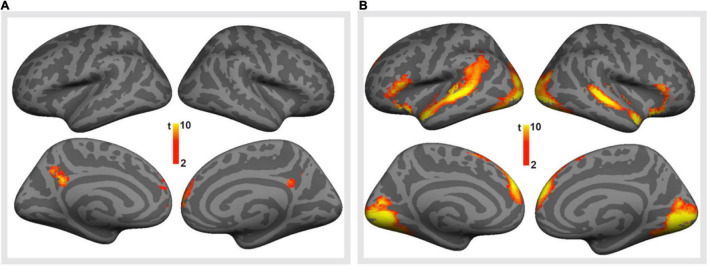
Brain responses to emotionally valenced vs. neutral stimuli. **(A)** Brain regions showing significantly stronger activity in response to viewing controversial (valenced) statements than to viewing neutral statements. **(B)** Brain regions showing on average significantly stronger activity in response to positive or negative peer feedback (i.e., valenced feedback) than to neutral feedback. Voxel-level family-wise error corrected *p* < 0.05, cluster size > 100 voxels for both.

We first studied brain responses elicited by the emotionally valenced stimuli and peer feedback (for cluster statistics, see Table 4). To this end, we compared the trials containing valenced statements with the trials containing neutral statements (Figure 3A; for the opposite contrasts, see Supplementary Figures 1, 2 and Supplementary Table 1). This contrast revealed activation clusters in the MPFC and the precuneus extending to the posterior cingulate cortex (PCC). Further, the trials with valenced peer feedback were contrasted with the trials with neutral feedback (Figure 3B). This analysis revealed that peer feedback elicited activity bilaterally in the VLPFC and MPFC, as well as in the occipital cortex (OC) and superior temporal gyrus and sulcus (STG/STS).

**TABLE 4:** Cluster probabilities, cluster size, peak coordinates, and effect size (Cohen’s d), for the whole-brain results.

Contrast	Cluster (Hemisphere)	Cluster probability	Size	Peak coordinate	Peak effect size
Valenced vs. neutral statements	MPFC (Both)	<1e^–16^	300	6,59,14	0.81
	Precun (Both)	<1e^–16^	166	−9,−52,32	0.93
Valenced vs. neutral feedback	MPFC (Both)	<1e^–16^	1254	−6,53,32	1.43
	OC (Both)	<1e^–16^	3320	0,−76,5	1.37
	STG/STS (LH)	<1e^–16^	1118	−57,−25,−1	1.44
	STG/STS (RH)	<1e^–16^	592	48,−28,−1	1.27
Negative vs. positive feedback	VLPFC (LH)	3.7e^–5^	206	−36,26,2	0.53
	VLPFC (RH)	0.029	107	30,20,−13	0.55
	SFG (LH)	0.0032	105	−6,50, 23	0.55
Positive vs. Negative feedback	pIns (LH)	2.7e^–4^	158	−27,−10,32	0.52
	TPJ (RH)	<1e^–16^	1543	39,−61,53	0.72
	SPL (LH)	1.6e^–4^	171	−51,−40,50	0.46
	SPL (RH)	3.4e^–4^	153	6,−34,38	0.58
	SFG (RH)	4.4e^–6^	262	27,17,53	0.62

*MPFC, medial prefrontal cortex; Precun, precuneus; OC, occipital cortex; STG/STS, superior temporal cortex; VLPFC, ventrolateral prefrontal cortex; SFG, superior frontal gyrus; pIns, posterior insula; TPJ, temporoparietal junction; SPL, superior parietal lobule.*

**FIGURE 4:**
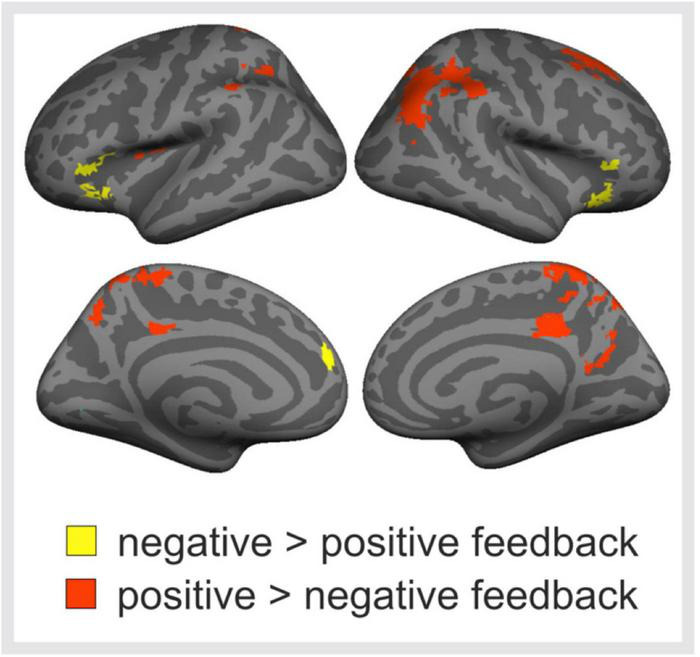
Brain responses to negative vs. positive peer feedback. Brain regions showing significantly higher activity in response to negative than positive peer feedback (yellow areas), and vice versa (red areas). Cluster-level family-wise error corrected *p* < 0.05, cluster size > 100 voxels.

Next, brain activations to positive and negative peer feedback were compared with each other (Figure 4). The results revealed that negative feedback was associated with significantly higher activity than positively valenced feedback bilaterally in the VLPFC, this activity extending to the anterior insula, and in the MPFC of the left hemisphere. Positive feedback, in turn social media use variables, was associated with significantly higher activity in the left posterior insula, around the temporoparietal junction (TPJ, i.e., the postcentral and angular gyri) especially in the right hemisphere, bilaterally in the medial superior parietal lobule, this activity extending to the precuneus and PCC, and in the superior frontal gyrus (SFG) of the right hemisphere.

#### Region-of-Interest (ROI) Analyses

The contrast between valenced peer feedback and neutral feedback (Figure 3B) was used to define seven ROIs: *Superior temporal, Inferior frontal*, and *Medial frontal ROIs* separately in the left and right hemisphere, and a bilateral *Occipital ROI*. Subsequent analyses were then restricted to voxels within these ROIS. To study individual differences in sensitivity to negative peer feedback, mean signal changes were then calculated for the contrast *negative feedback > positive feedback* and subjected to mixed ANOVAs.

In the first mixed ANOVA with ROI as the within-participant variable and the social media use measures as between-participant variables (covariates age and gender), there was no significant main effect of any of the three social media use variables (all *p*-values > 0.26). The social media use variables did not interact significantly with the ROI factor, either (all *p*-values > 0.31 to 0.56).

## Discussion

### Brain Responses to Emotion-Evoking Stimuli and Peer Feedback

The present social media task utilized emotion-evoking stimuli both in the form of controversial statements, as well as negative and positive peer feedback. When examining neural responses to statements that participants were asked to evaluate and respond to, a network of medial cortical regions was observed to respond to their emotional content. Regions located in the precuneus, PCC, and MPFC responded more strongly to controversial statements than to their neutral counterparts. These cortical midline structures have been suggested to be involved in internally oriented attention and self-referential thinking (Whitfield-Gabrieli et al., 2011; Leech and Sharp, 2014; Davey et al., 2016). Importantly, activity in these regions has repeatedly been linked to decision-making and judgment that requires self-reference (Feyers et al., 2010; van der Meer et al., 2010; Denny et al., 2012; Fede and Kiehl, 2019). The present findings, therefore, concur the role of cortical midline regions in real-time decision-making. More specifically, our results suggest that these regions might be especially important when decisions are made on emotion-evoking material in a social context, since judgments concerning the accuracy of neutral facts did not involve the midline network to the same extent.

Valanced peer feedback activated a wide-spread network of brain regions. Peer feedback was associated with enhanced activity in the anterior MPFC in both hemispheres. This finding is of special interest, as the anterior MPFC regions have been implicated in social cognition tasks involving self-knowledge, person perception, and mentalizing (for reviews, see Amodio and Frith, 2006; Bzdok et al., 2012). Frontal midline structures, in general, have been linked to internally oriented mental activity (Buckner et al., 2008; Dixon et al., 2014), for example, during self-referential processing (Northoff et al., 2006; Kim, 2012) and self- and other-oriented evaluation (Araujo et al., 2013). It is therefore possible that receiving feedback on one’s opinions encourages self-reflection or mentalizing, or both, to more carefully analyze the motives and meaning behind the feedback.

In addition to the MPFC activity, clusters of activity were seen bilaterally in the OC and STG/STS. We did not expect to observe responsivity in these areas to peer feedback, as they are most often linked to perceptual processing of visual and auditory stimuli, respectively. Heightened activity in these areas might, however, reflect enhanced attention to the emotionally engaging and socially relevant peer feedback, as attention is known to modulate activity in the sensory cortices (Woodruff et al., 1996; Johnson and Zatorre, 2006; Alho et al., 2016). Yet, as no auditory stimuli were used in the present paradigm, the enhanced activity in the STG/STS might be partly due to more careful processing of the verbal content of emotional than neutral written feedback, as this region is known to be involved in language comprehension (Parker et al., 2005; Friederici, 2012) also during reading (Choi et al., 2014; Evans et al., 2016). This is supported by the fact that heightened activity was also observed in the inferior frontal gyrus especially in the left hemisphere, another region involved in linguistic comprehension (Friederici et al., 2003; Vigneau et al., 2006; Moisala et al., 2015). However, the STG/STS has not only been linked to language processing but also to social cognition: theorizing about other minds (Bzdok et al., 2012), detecting socially salient stimuli (Adolphs, 2003) and processing varied other forms of social information (Deen et al., 2015). It is, therefore, possible that the strong STG/STS activity to emotionally valenced feedback was observed in the present study because the peer feedback was perceived as socially salient, and because it required perspective-taking from the part of the participants. Further studies are needed to tease apart these possible explanations for the activations seen in temporal regions.

The current study utilized an experimental task that was developed to mimic real-life adolescent interaction in an authentic social media (Facebook) environment. Participants’ reactions before debriefing suggested that the task had been credible. Our main findings regarding brain activity elicited by virtual peer feedback were similar to those obtained in studies with simpler experimental tasks. That is, in accordance with previous studies (e.g., the Cyberball task study of Gunther Moor et al. (2012) and the study of Achterberg et al. (2016) where profile pictures were rated), we also observed heightened mPFC activity in response to emotionally valenced peer feedback. Thus, our findings with a more naturalistic task than in previous studies replicate the findings of more controlled albeit less naturalistic paradigms, encouraging researchers studying peer feedback and rejection to develop increasingly more ecologically valid tasks and utilize existing real-life social platforms.

### Differences in Brain Responses to Negative and Positive Peer Feedback

Further analyses of the present brain imaging data revealed some differences between neural responses to positive and negative peer feedback. Expectedly, negatively valenced peer feedback activated the VLPFC and anterior insula more strongly than positive feedback, and activity was also seen in the MPFC. These findings are consistent with previous research on adolescent peer rejection. Masten et al. (2009) found that VLPFC activity during social exclusion correlated negatively with subjective feelings of distress, causing them to conclude that VLPFC functioning was likely related to emotion regulation. The VLPFC has been linked to cognitive control (Ryman et al., 2019), affective appraisal of valenced peer feedback (Guyer et al., 2012), and emotion regulation (Riva et al., 2015), and thus, it is possible that negative feedback, in particular, requires control over one’s emotional reaction.

Masten et al. (2009) also observed engagement of the anterior insula during social exclusion, a finding replicated more recently (Cacioppo et al., 2013; Achterberg et al., 2016). Anterior insular activity has been linked to experiencing both physical pain as well as emotional pain (Eisenberger et al., 2003; Kross et al., 2011; Orenius et al., 2017), but the engagement of the anterior insula during emotion-evoking tasks may also be due to a more generic responsivity to salient events (Gasquoine, 2014). Taken together, the observed recruitment of the anterior insula and VLPFC during negative feedback in the current study likely reflects an elevated negative affective state or level of arousal, coupled with an increased need for emotion regulation.

Contrary to our expectations, we did not observe any ACC activity in response to negative feedback. Previous studies have linked this region to social and emotional evaluation and affective distress in experimental tasks including social rejection (Gunther Moor et al., 2012; Cheng et al., 2020) or peer feedback (Somerville et al., 2006; see, however, Tan et al., 2014). Perhaps the tasks in the previous studies were able to elicit stronger emotional reactions and involve the ACC more strongly than the present task, as one could argue that being socially rejected elicits stronger negative emotions than getting negative peer feedback. Also, previous studies (e.g., Goldstein et al., 2010) have shown that females and males differ in their ACC responsivity to negative valanced stimuli, with female participants showing high variability in their ACC responsivity. Therefore, it may be that such variability in the current data (over half of the participants were female) masked possible effects in the ACC. It should be also noted that in the present study, the probability of “peer rejection” (i.e., negative feedback) was higher than the probability of peer rejection in the Cyberball studies that showed enhanced activity in response to peer rejection both in the ACC and insula (Gunther Moor et al., 2012; Cacioppo et al., 2013). In the Cyberball paradigm, exclusion occurs rather unexpectedly, which might partly explain the dorsal ACC and anterior insula involvement as a salience network. In the present study, however, there was an equal number of positive and negative feedback conditions, and therefore negative feedback in the present study was less unexpected than peer rejection in the Cyberball paradigm.

Several brain regions demonstrated greater activity in response to positive than negative feedback in the current study. These regions have been related previously to impulse control (the SFG; Dambacher et al., 2014), self-referential processing (the PCC and precuneus; Whitfield-Gabrieli et al., 2011; Davey et al., 2016), and theorizing about other minds (the TPJ; Saxe and Kanwisher, 2003). A similar set of regions have been shown to be involved in social cognition both during peer presence and evaluation by peers (van Hoorn et al., 2016). The temporo-parietal brain regions were also found to be involved in prosocial decision-making during interactions with friends (Schreuders et al., 2018, 2019). Speculatively, this activation pattern was interpreted to suggest that posterior parietal brain regions might support spontaneous integration of self- and other-related perspectives that arise from positive peer interactions (Carter and Huettel, 2013). The current findings further extend the involvement of these brain regions of social cognition to positive feedback from anonymous others. Notably, TPJ was also involved in processing of positive feedbacks based on a personal interview (van Schie et al., 2018). Moreover, this neural response to positive feedback was shown to be related positively to self-esteem. These findings pave the way for future research that should aim to investigate how positive feedback received during online interactions might relate to self-esteem across adolescence.

### Associations Between Sensitivity to Negative Peer Feedback and Individual Differences Social Media Use

In the current study, individual differences in social media use were also investigated to determine whether these factors demonstrate associations with neural reactivity to virtual social rejection. When the social media use variables were examined, no association was found with brain responses to negative feedback, suggesting that social media use activity is not strongly reflected in responsivity to negative social feedback on a neural level. In order to determine whether responding more strongly to virtual social rejection is a major driving force behind how active adolescents are on social media, future studies would benefit from using more sensitive measures of real-life social media use than self-report questionnaires. Other measures apart from brain imaging could also be used to measure emotional reactivity (e.g., recordings of facial expressions), and emotional reactivity to negative and positive virtual peer feedback could be more carefully tracked in real-life situations outside of the laboratory environment. It is important to study more carefully the driving forces behind young people’s social media use since individual levels of daily virtual social interaction are linked to academic engagement and wellbeing (Hietajärvi et al., 2019), as well as a host of mental health outcomes (e.g., Barry et al., 2017; Kelly et al., 2018).

### Limitations of the Present Study

Previous studies using quite simple paradigms may not have captured the full extent of neural networks involved in real-life (online) social interactions. Based on the results from our current, more ecologically valid paradigm, we show that the same brain networks activated in previous studies utilizing simpler paradigms, are also involved in more complex social interactions in virtual real-life situations. However, our use of a more complex, realistic, and ecologically valid paradigm also has some potential down-sides. For example, an interview of participants after the experiment could help to verify the cognitive, affective, and social processes they were engaged in during the task so that these processes could be linked more precisely to the participants’ measured brain activity. Moreover, although the present experiment aimed at mimicking a Facebook environment, the preregistered feedback responses and their pre-set presentation rate limited the ecological validity of social interaction in the present study. Therefore, the results obtained should be replicated in future brain imaging studies applying social media interaction without these technical constraints.

Furthermore, our control task included factual neutral statements that were all correct and required the participants to reply either “true” or “false,” and the participant’s response were followed by further factual neutral statements. This task was used to elicit visual, linguistic, and motor processes similar to those activated in the main task. This may be problematic, since the participants found most of these neutral statements to be “true,” when in the main task, they agreed with only about half of the controversial statements. Therefore, there may have been differences in cognitive decision-making processes between the main and control tasks. Thus, brain activity associated with these processes may have contributed to the brain responses revealed by contrasting the controversial statements with the neutral statements (Figure 3A). These differences may have even carried over to brain activity comparisons between valenced (positive or negative) feedback following controversial statements and neutral feedback (statements) following factual neutral statements (Figure 3B). However, we assume that the brain activity differences revealed by these comparisons were mostly due to the socio-emotional processing of the controversial statements and the valenced feedback, since these activations did not include the dorsal prefrontal and superior parietal areas typically activated during emotionally neutral (e.g., perceptual or linguistic) decision-making tasks (see e.g., Keuken et al., 2014; Moisala et al., 2015).

Finally, social cognition and emotions, as well as the underlying brain functions, are strongly connected (e.g., Adolphs, 2003; Olsson and Ochsner, 2008; Niedenthal and Brauer, 2012). Due to this, and due to emotions possibly elicited by socially controversial statements and the affective valence of peer feedback, it is not possible to resolve whether the brain activations observed in the present study are related to emotions or social cognition, or both.

Even given its limitations, the present study provides new insight into the relationship between technology use and brain function in several ways. This study was able to demonstrate that using a more naturalistic experimental task was still able to produce robust activations in similar brain regions as in previous studies that have used more simplistic experimental stimuli, thus bringing us one step closer to understanding how the social brain works during real-life social media use. Also, the fact that there were no significant associations between social media use variables and neural activation is a valuable finding in itself (however, make note that these analyses were slightly underpowered in the current study, see section “Participants and Procedure”). This suggests that neural responsivity to emotionally valenced social feedback might not strongly explain variability in daily social media use. Instead, other factors, for example, addiction vulnerability or lack of inhibition, might play a more important role. However, to clarify this, further studies addressing these factors are needed.

## Conclusion

The results of the current study show that responses in the social brain network (e.g., VLPFC, MPFC, and STG/STS) in late adolescence and early adulthood to peer feedback in social media vary according to the valence of the feedback. Self-reported habits of social media use did not correlate with brain responses to negative feedback, indicating that future studies would benefit from using more sensitive and objective measures of real-life social media use than self-report questionnaires. By utilizing a task mimicking social media use, the present study provides new insight into the relationships between technology use and brain function. Further investigating the neural mechanisms behind the emotions elicited by virtual peer interaction may help us understand why adolescents and young adults are so intensively involved with social media platforms in their daily lives.

## Data Availability Statement

The datasets presented in this article are not readily available because ethical approval does not permit. Requests to access the datasets should be directed to MM.

## Ethics Statement

The studies involving human participants were reviewed and approved by the Ethics Committee of The Hospital District of Helsinki and Uusimaa. Written informed consent to participate in this study was provided by the participants’ legal guardian/next of kin.

## Author Contributions

MM, PW, SL, KA, and BG designed the paradigm. MM, AY, JL, and PW collected the data. MM, PW, and JL analyzed the data. PW, MM, and KA wrote the initial draft of the manuscript. KL, KA, and KS-A applied for funding for the project. All authors edited the manuscript.

## Conflict of Interest

The authors declare that the research was conducted in the absence of any commercial or financial relationships that could be construed as a potential conflict of interest.

## Publisher’s Note

All claims expressed in this article are solely those of the authors and do not necessarily represent those of their affiliated organizations, or those of the publisher, the editors and the reviewers. Any product that may be evaluated in this article, or claim that may be made by its manufacturer, is not guaranteed or endorsed by the publisher.

## Footnotes

1
http://www.alivelearn.net/xjview

2
http://marsbar.sourceforge.net/
