# Supplementary material for: Brain Responses to Peer Feedback in Social Media Are Modulated by Valence in Late Adolescence
Source: Front Behav Neurosci. 2022 May 30;16:790478. doi: 10.3389/fnbeh.2022.790478 (PMC9190756; doi:10.3389/fnbeh.2022.790478)
Supplement: Supplementary file 1 [file Data_Sheet_1.docx]

| Supplementary Table 1. Cluster probabilities, cluster size, peak coordinates and effect size (Cohen’s d), for the whole brain results. AG: Angular gyrus, IFS: inferior frontal sulcus, PCC: posterior cingulate cortex | | | |  |  |
| --- | --- | --- | --- | --- | --- |
| Contrast | **Cluster (Hemisphere)** | **Cluster probability** | \| **Size** \| \| --- \| \| **10.42** \| \| **8.29** \| \| **6.05** \| \| **9.34** \| \| **11.02** \| \| **6.85** \| | **Peak coordinate** | **Peak**  **effect size** |
| Neutral vs. valenced statements | **AG (RH)** | **1.1e^-10^** | **383** | **39,-70,47** | **1.09** |
|  | **AG (LH)** | **6.1e^-8^** | **270** | **-30,-73,44** | **0.83** |
|  | **IFS (RH)** | **5.1e^-8^** | **274** | **48,35,20** | **0.70** |
|  | **IFS (LH)** | **1.2e^-4^** | **126** | **-45,35,17** | **0.61** |
| Neutral vs. valenced feedback | **AG (RH)** | **< 1e^-16^** | **126** | **45,-73,35** | **1.27** |
|  | **IFS (LH)** | **< 1e^-16^** | **209** | **-48,35,26** | **1.25** |
|  | **Pre (LH)** | **< 1e^-16^** | **110** | **36,26,2** | **1.17** |
|  | **Pre (LH)** | **< 1e^-16^** | **112** | **12,-55,20** | **1.15** |
|  | **AG (LH)** | **< 1e^-16^** | **167** | **-39,76,32** | **1.10** |
|  | **PCC (Both)** | **< 1e^-16^** | **251** | **6,-34,44** | **1.08** |
|  | **SFG (RH)** | **< 1e^-16^** | **127** | **24,14,53** | **1.07** |
|  | **SFG (LH)** | **< 1e^-16^** | **120** | **-24,11,56** | **1.05** |
|  | **IPL (RH)** | **< 1e^-16^** | **638** | **60,-34,44** | **1.04** |
|  | **IPL (LH)** | **< 1e^-16^** | **198** | **-54,-40,53** | **0.96** |
|  | **Ins (RH)** | **< 1e^-16^** | **180** | **-42,-1,8** | **0.91** |

**Supplementary Table 2.** Translated statements used in this study

Neutral

Migratory birds fly to south in the autumn.

Finland is a member of the European Union.

Mandarin has the most speakers in the world.

The currency of Finland is euro.

Finland became independent in 1917.

Football is the most popular sport in the world.

Finns drink the most coffee in the world.

The brain is an important organ located in the head.

Chimpanzees are the animals most closely related to humans.

The capital of Sweden is Stockholm.

Finland has had a female president.

Dogs have been bred from wolves.

The bride’s wedding dress is often white.

Children learn to walk when they are about one year old.

Youtube is a website for sharing videos.

Human pregnancy lasts about 9 months.

Seasons are different across the world.

The Earth is part of the Milky Way.

The main ingredient of chocolate is cocoa.

The national composer of Finland is Jean Sibelius.

Valenced

Death sentences should be allowed in Finland.

The voting age should be lowered to 16 years.

A 16-year-old should already be eligible for a driver's license.

Immigration should be restricted in Finland.

Poverty is one's own fault.

Mothers are closer to their children than fathers.

Men are more intelligent than women.

Having children is selfish.

God created the universe.

In Finland, people pay too much taxes.

Students should be paid more subsidies.

University studies should be subject to charge.

Men are naturally better leaders.

Social security should be cut.

Environmental protection is important.

Global warming should be stopped.

Fur farming is unethical.

People should eat less meat.

Harassers should be punished more severely.

Euthanasia should be legal.

Alcoholism is a disease.

Depression should be treated with therapy, not drugs.

Small babies should not be allowed in fancy restaurants.

People who chew with their mouths open are annoying.

Learning Swedish should not be compulsory.

Already in the upper secondary school, one should be free to study only what one pleases.

Getting into a university is too difficult in Finland.

Life experience is more important than education.

Intelligence is innate.

Income gaps are due to differences in industriousness.

Obesity is due to laziness.

Sweets should be taxed more.

Schools should exercise stricter discipline.

Physical discipline is acceptable in some situations.

Abortion should be illegal.

**Supplementary Table 3.** Correlations (Pearson’s bivariate) between the activity in each ROI and the extraversion and agreeableness.

|  | left MPFC | right MPFC | left IFG | right IFG | left STG/S | right STG/S | OCC |
| --- | --- | --- | --- | --- | --- | --- | --- |
| Extraversion | .10 | .09 | .06 | -.04 | .09 | .04 | .07 |
| Agreeableness | .22 | .20 | .20 | .24 | .22 | .17 | .22 |


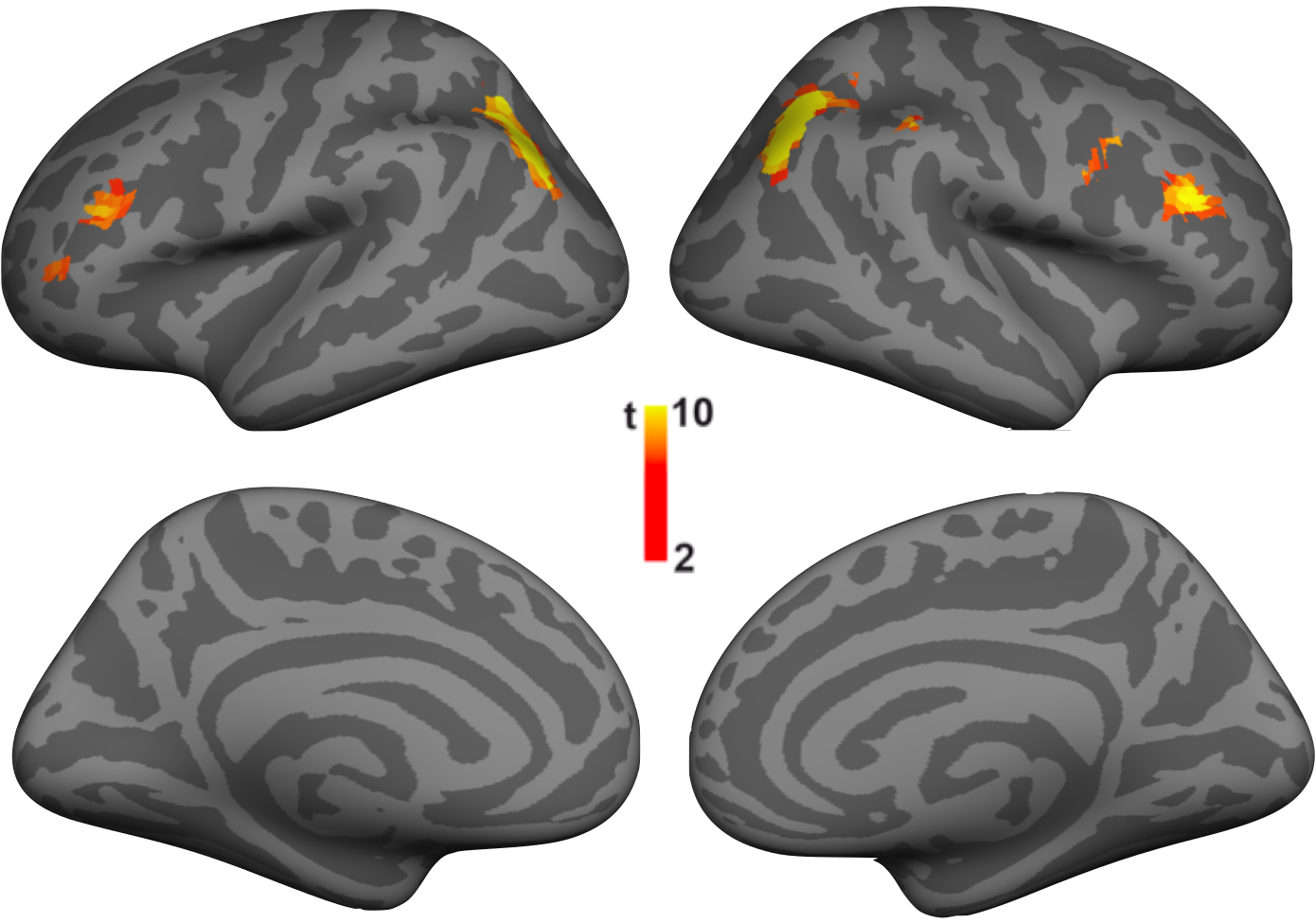


**Supplementary Figure 1. *Brain responses to emotionally neutral vs. valenced statements.*** *Brain regions showing significantly stronger activity in response to viewing neutral statements than to viewing controversial statements****.*** *Voxel-level family-wise error corrected p < 0.05, cluster size > 100.*

*
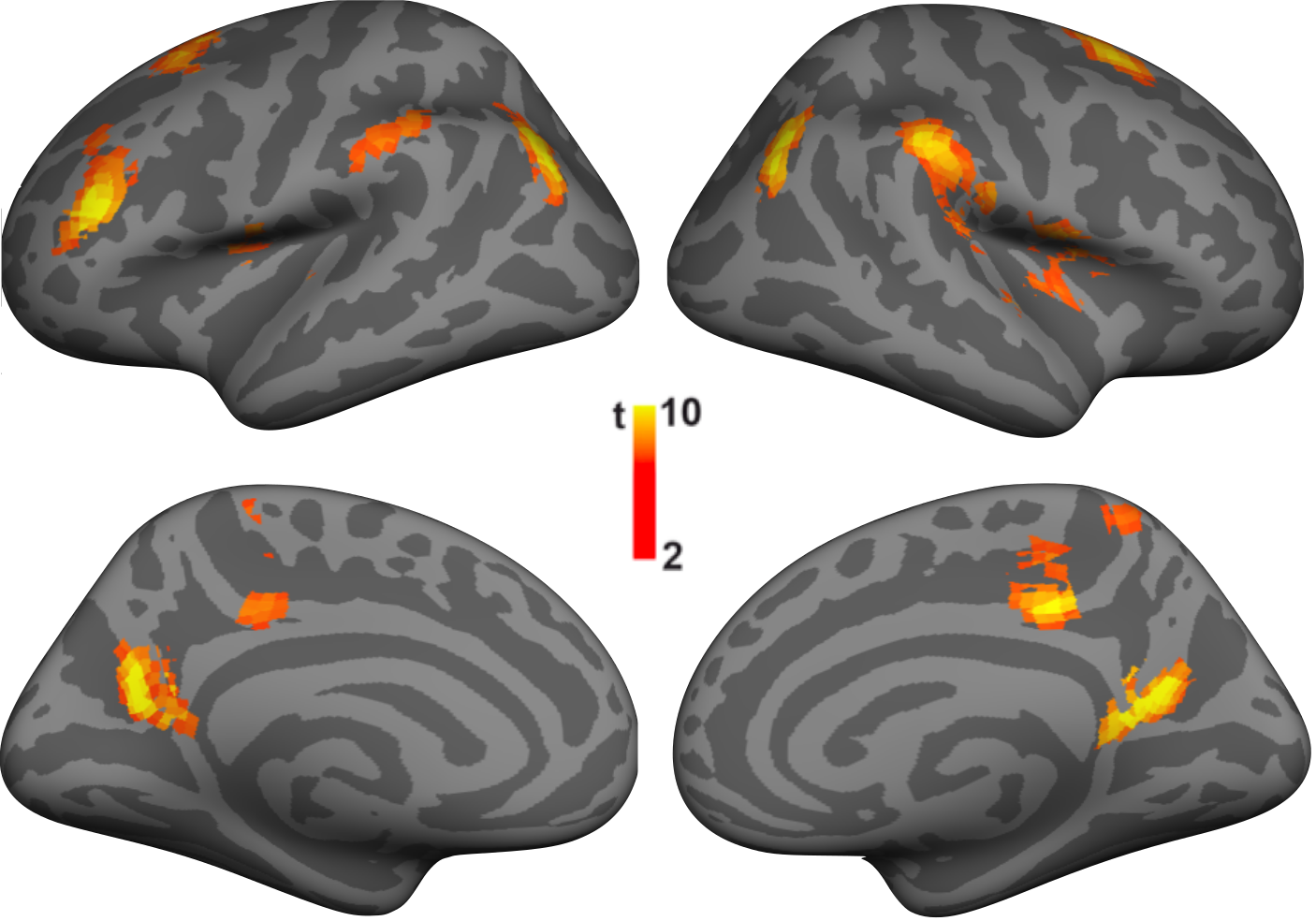
*

**Supplementary Figure 2. *Brain responses to emotionally neutral vs. valenced feedback.*** *Brain regions showing significantly stronger activity in response to viewing neutral feedbacks than to viewing controversial feedback****.*** *Voxel-level family-wise error corrected p < 0.05, cluster size > 100.*
